# Embryo Toxic Effects of Depleted Uranium on the Morphology of the Mouse Fetus

**Published:** 2014

**Authors:** Nina Mirderikvand, Baharak Mohammadzadeh Asl, Parvaneh Naserzadeh, Fatemeh Shaki, Mohammad Shokrzadeh, Jalal Pourahmad

**Affiliations:** a *Department of Pharmacology and Toxicology, Faculty of Pharmacy, Shahid Beheshti University of Medical Sciences, Tehran, Iran. *; b *Department of Pharmacology and Toxicology, School of Pharmacy, Mazandaran University of Medical Sciences, Sari, Iran.*

**Keywords:** Depleted uranium, Morphology, Mouse fetus, Embryotoxicity

## Abstract

Although the biokinetics, metabolism, and chemical toxicity of uranium are well known, until recently little attention was paid to the potential toxic effects of uranium on reproduction and development in mammals. In recent years, it has been shown that uranium is a developmental toxicant when given orally or subcutaneously (SC) to mice. Decreased fertility, embryo/fetal toxicity including teratogenicity, and reduced growth of the offspring have been observed following uranium exposure at different gestation periods. For investigating the effects of DU on pregnant animals, three groups (control, sham and test) of NMRI mice were chosen. In test group 4 mg/Kg of DU were administered intraperitonealy at 11 day of gestation, in sham group only normal saline injected to interior peritoneum as indicated in the test group and in Control group which was considered as the comparison base line of our research, no injection was made. Caesarean sections were performed at 15 day of the gestation; and their placentas were examined externally. Base on our results DU caused significant external anomalies, and caused a significant decrease (p < 0.05) in the weight and diameter of placentas, the number of the embryos, their body weight and crown-rump length of fetuses.

## Introduction

Uranium (U) is a naturally occurring element the best known use of which in the last 70 years has been as fuel in nuclear power reactors and nuclear weapons (1). During uranium processing, workers may inhale or ingest some uranium giving rise to internal contamination, which could result in radiation doses to the body (2, 3). In addition, if uranium exposure were large enough, chemical toxicity could also occur. Under some circumstances, the chemical toxicity of soluble uranium compounds can even surpass the potential radiotoxic effects. The general population may be exposed to low levels of uranium by inhalation or through the diet. Uranium may be also introduced into drinking water supplies through the mining and milling of uranium ore. The principal objectives included the establishment of exposure limits for airborne uranium in the workplace based upon uranium’s known chemical renal damage. Although the biokinetics, metabolism, and chemical toxicity of uranium including the toxic effects of this metal on kidney function, are well established, until recently there was a lack of published observations regarding uranium-induced reproductive and developmental toxic effects (4, 5).

Uranium, atomic number 92, is a naturally occurring element. The best known use of uranium is as a fuel in nuclear power reactors. Due to its important role in the nuclear industry, a considerable research effort dealing with the metabolism of uranium in mammals has been prompted during the last decades (6) Penetration of uranium into the animal body may be accomplished by several routes: intravenous, Intraperitoneal, inhalation, oral, sub coetaneous, *etc*. 

According to the latest information in the database there are only a few articles which have studied the toxicity effects of uranium on fetuses or teratogenicity of this metal on mammals.

In one of these studies, doses of 0, 5, 10, 25 and 50 mg/Kg/day of Uranium acetate dehydrate was taken to pregnant mice during the pregnancy period of 15-6 days.

Toxic effects have been reported as weight loss, reduced food intake and growth retardation in pregnant mice that did not depend on the effects of uranium toxic doses, but doses by more than 5 mg/Kg/day were caused toxicity in embryos. Cleft palate, hematoma, loss of bone in the skull of the fetus were the effects of uranium toxicity which is reported in this study (7). In another study, maternal toxicity, reduced weight and increased relative liver weight was observed. Fetal toxicity consisted of decreased fetal body weight, body length, cleft palate, and mass region of blood on face was seen. Problems such as rotation and reduced ossification of skull and bones, tail, hind foot and paragraphs Metatarsus the front toes were exist (8).

There is only an available article which is related to the direct effects of Depleted Uranium in the mouse embryo. In this study the Depleted Uranium with different concentrations was implanted in the body of pregnant rats, the female mice were mated and pregnant, at twentieth day of pregnancy the embryo were taken outside the body of all pregnant mice the level of Uranium in liver, kidney and fetal plasma were measured. Although uranium levels were increased in fetal body, but no specific toxicity such as weight loss, and small size were observed (9).

As “Bertel” mentioned in Teratogenic Toxicity: “Soluble Uranium oxide and Nano-particles can cross the placenta to the fetus and rapidly toxic the growing fetus. At low doses, Nano-particles damage the fetal brain and cause mental retardation and behavioral problems. Other teratogenic effects include various deformities and diseases, and even affect the growth hormone and the immune system of fetal. In the research was carried out male rats, were received Uranium acetate dehydrate with doses (0, 5, 10 and 25 mg/Kg day) by gavages for 60 days then mated with females that had been treated for 14 days. Growth and development of infants were studied after 0, 4 and 21 days of lactation; in this period the increases in the number of dead infants were observed. (10).

The potential toxicity or adverse effects of DU in pregnant women who were exposed to depleted uranium. in the recent 60 years was including; 1 - Female victims of using banned bombing weapons containing depleted uranium in countries such as Iraq, Kuwait, Afghanistan). 2 - Women employed in civilian nuclear reactors, uranium mines and mining. 3- Women who participate in the process of nuclear waste or living near a nuclear waste disposal site, was always one of the most serious concerns’ in environmental toxicology. 

Possibly Contact with depleted uranium leads to toxicity and fetal malformations in the fetus and finally repeated abortions in pregnant mice. Therefore, toxicological and pathological we search to determine the effects of Depleted Uranium in embryonic cells and mitochondrial damage in mouse embryonic cells will be more than necessary in our country which is going to use peaceful nuclear energy, outcomes of this research will provide protective strategies which can counter the toxic effects of depleted uranium in high risk women, and also arrangements for proper disposal of the industrial waste, including depleted uranium.

## Experimental

*Devices and tools *

Binocular optical microscope, Research Microscope Model M3, Manual rotary microtome, One scale balance with an accuracy of 0.1 g, Analytical balance with an accuracy 0.0001 g, Water distillation apparatus, Loop binoculars, Timer, Surgical Sets (forceps, scalpel, scissors, dissection tray *etc*), Laboratory glassware (flask, graduated cylinders, desiccators *etc*), Molding Wax for molding glass, Tissue warming plate, The Oven, Metal cages for keeping the animals, Computer and printer, slide and cover slip, Filter Paper, Digital camera, Latex Gloves, Cotton hydrophilic

*Compounds*

Uranyl acetate dehydrate, Medical alcohol, Toluene, Paraffin with a melting point of 60-56 °C, antler glue, xylene, Picric acid, bouin's fixative solution, Haupt's gelatin, Glacial acetic acid, Formalin, Haematoxylin, Eosin powder, Gelatin, Ether, were purchased from Sigma–Aldrich Co. (Taufkrichen, Germany). All other chemicals were of the highest commercial grade available. Normal saline and Distilled Water were offered as a generous gift by Daroo Pakhsh Co.Ltd.Tehran, Iran.

*Animal*

The animal used in this research, were mice of NMRI race, purchased from Institute Pasteur (Tehran, Iran). Firstly, the limited numbers of healthy mice separated, their feeding and housing have done in special cages which their floor covered by woody chips. The food consisted of ready pallets. Their necessary water supplied by special bottles. In order to preventing any kind of pollution, the woody chips in floor of cage changed at least once a week and their consuming water bottle replaced 3 or 4 times in this period of time. Every few days, the cages disinfected by Formalin 6%, ethanol or liquid bleach. The temperature and moisture of the room determined regularly. All mice were housed in a room at a constant temperature of 25 ºC on a 12/12 hr light/dark cycle with food and water available. All experiments were conducted according to ethical standards and protocols approved by the Committee of Animal Experimentation of Shahid Beheshti University of Medical Sciences, Tehran, Iran. The ethical standards were based on “European Convention for the Protection of Vertebrate Animals Used for Test and other Scientific Purposes” Acts of 1986, and the “Guiding Principles in the Use of Animals in Toxicology,” adopted by the Society of Toxicology in 1989, for the acceptable use of test animals. All animals received humane care according to the criteria outlined in the “Guide for the Care and Use of Laboratory Animals” prepared by the National Academy of Sciences and published by the National Institutes of Health (NIH publication 86 – 23, revised 1985).The mice has monitored during the study period, the weak and sick mice has distinguished and eliminated. In this study, in order to investigate the effect of uranyl acetate dihydrate on growth of mice fetus, 3 groups each contained 6 female adult mice selected. Before that, the pregnant mice were chosen by observing the vaginal smear and plug. In this project following groups were considered and studied:

1. Test group: in this group half of 6 adult female mice which did not have the experience of mating have been selected. The uranyl acetate dihydrate with the concentration of 4 mg/Kg/day injected intraperitonealy. This solution injected at 11 day of gestation.
2. Sham group: in this group, only normal saline injected to interior peritoneum as indicated in the test group.
3. Control group: this group which was consider as the comparison base line grew up in a quite normal condition.

The animals were anesthetized at 15 day of gestation. Following laparatomy, the uterus was exteriorized and the number and location of fetuses and resorption were noted, then their weight and length (crown- rump length) were measured. Individual fetuses were examined carefully for external anomalies then fetuses were stained by haematoxylin-eosin method and investigated by stereomicroscope for skeletal malformations. The incidence of skeletal malformations and other histological lesions were determined and compared in the groups. Statistical significance between groups was determined using SPSS program and compared by one way analysis of variance (ANOVA).

Binomial data were examined using the Chi-square test. The minimum level of significance was p < 0.05.

*The surgery and tissue preparation*

Mice were supine in description tray after being anesthetized by ether or chloroform. The uterus including fetuses and placenta are visible after opening abdominal skin in front of the vagina. The tissue sections were prepared in series in order to investigate the microscopic variations due to toxic effect of uranyl acetate dihydrate on ovary. In the following, the different stages of preparation were summarized.

*a) Tissue fixation*

The tissue samples should be fixed for preparing to maintain their natural shape and avoid marked alterations. Leaving tissues in the air may causes evaporation and this water loss may cause shrinkage. On the other hand, putting the samples in water causes swelling or osmotic pressure.

In order to fix tissues obtained from fetuses, we rinsed them by physiologic serum and put them in bouin's fixative solution for 18 hours then we continue with dehydrating stages.

*b) Dehydration, Clearing, Infiltration*

The process of removing cellular and tissues water is called dehydrating which proceed by medicinal ethyl alcohol. After dehydrating the alcohol replaced with cellular and tissues water should be extracted. Toluene is used for extracting alcohol. After clearing stage we introduce paraffin in to tissues that this procedure was done by inserting tissues in the paraffin bath and oven (11).

*c) Embedding*

Once tissues saturated completely by paraffin we started embedding process. For this purpose the samples were taken out from paraffin bath 2 and put it into glassy molds which are specified for embedding and contains melt paraffin. This stage should be done immediately and carefully in order to prevent bubble formation. Besides the samples should be located in the middle of the mold (minimum distance of 1mm from bottom) (11).

*d) Sectioning*

Before cutting, the molds contain paraffin were shaped in to small trapezoid. In other word, the molds were shaped in to neat and regularized forms, we call this procedure trimming. Then the molds are fixed in special tray for installing on microtome. After fixing molds on microtome and sectioning, the paraffinic bands containing tissue sections with thickness of 10 micrometer for fetus and 6 micrometer for placenta, uterus, and ovaries, were achieved in series.

*e) Sticking the paraffinic bands on slides*

The obtained paraffinic bands were transferred on slides which covered by Haupt's gelatin glue. In order to eliminate the shrinks on paraffinic bands, the surface of slides should already be covered with formalin 2%. We put paraffinic bands to float on these slides and finally, the slides were located on warming plate which its heat is adjusted to 40 °C (10-15 °C) lower than the melting point of paraffin). After complete spreading and drying the sample, we took the slides from warming plate.

*Staining*

Not only the nuclei and cytoplasm of cells but also the connective tissues surrounding the cells were stained. The staining procedure performed as follows (Table 1).

**Table 1:** Time table of sequences of events in the staining procedure

**Sequence of event**	**Stage**	**Material**	**Time**
1	Dewaxing of paraffin	Toluene	5-6 minutes (if necessary repeated)
2	Hydration	Alcohol 100 Alcohol 90 Alcohol 70 Alcohol 50 Distilled water	5 min 5 min 5 min 5 min 5 min
**3**	Staining	Haematoxylin Washing with water Alcohol 50 Alcohol 70 Eosin	3-5 min 5 min 5 min 5 min 5 min
4	Dehydration	Alcohol 90 Absolute Alcohol	30 second 30 second
**5**	Clearing	Toluene	5-10 minutes

*Mounting the cover slips*

As soon as finishing the staining process and before drying the samples, we stuck the samples with antler glue on slides. This procedure should be done carefully in order to preventing the bubble formation on the slides. Then the cover slips were mounted on slides.

*The investigation on tissues*

The stained sections were investigated microscopically and the microscopic parameters of fetuses belong to sham, test and control group were compared. Parameters compared include weight of mother and fetus, weight of placenta (measured by digital balance), placenta diameter and size of fetus C-R (Crown - Rump Length) (measured microscopically by caliper).

## Results and Discussion

In this study the teratogenicity of depleted Uranium on NMRI mouse fetus was studied with, after appropriate tests the results of this research were demonstrated and discussed along with findings of previous similar works. In the morphological and macroscopic point of view the things that seemed to be noteworthy in this research were that liver of fetuses in test group were bulkier and head of them also were larger than those of control and Sham groups (pictures not shown).

*Histological results*

**Figure 1:**
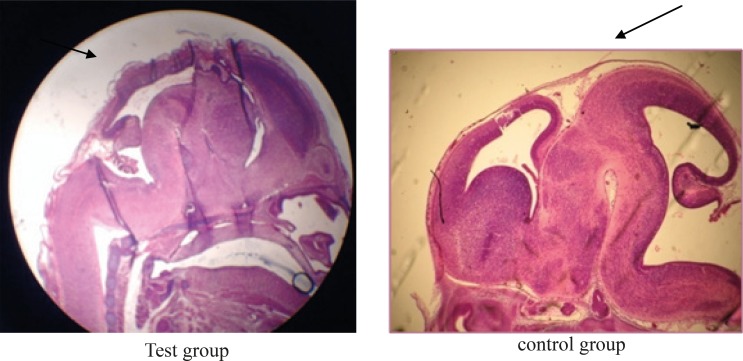
Comparison of the curves in the cerebral cortex of fetuses in test and control groups.

**Figure 2:**
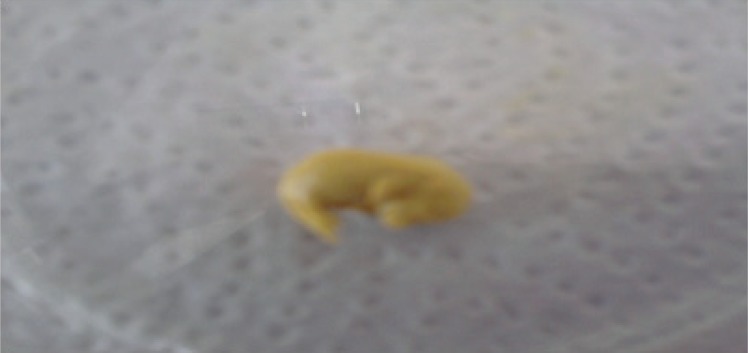
The lack of fingers in the test group

**Figure 3:**
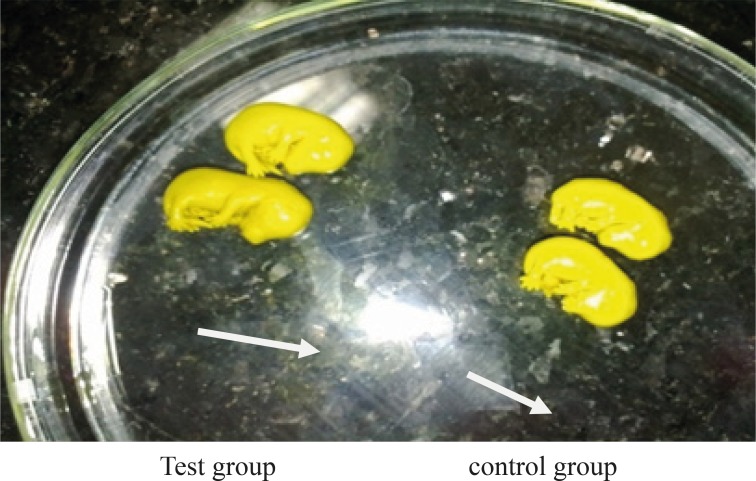
Comparison of the correct rotation of fetus and the CR in both test and control groups

**Figure 4:**
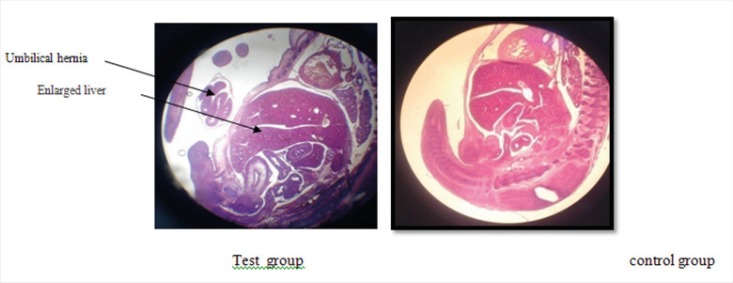
View of enlarged liver and also umbilical hernia in the test group due to the delay in the fetal development

The photomicrographs were prepared from samples of test, Sham, and control groups which some of them are shown in pictures 1, 2, 3 and 4. The important findings in test group compare to sham and control groups were:

1. Lack of specific curve in cerebral cortex (Figure 1).
2. In one case the fetus did not have fingers in hand (Figure 2).
3. The rotation of C shape in fetus of test group did not observe and their backs were straight (Figure 3).
4. The weight of fetuses and the size C-R were more than control group (Figure 3).
5. The appearance of liver was large and dark (Figure 4).
6. Disappearing of umbilical hernia in 15 days fetus is the reason of delay in growth of it (Figure 4).

*Alteration in the weight of fetuses*

The total weight of fetuses in test group were significantly (P < 0.001) increased compared to control and Sham groups .

**Figure 5:**
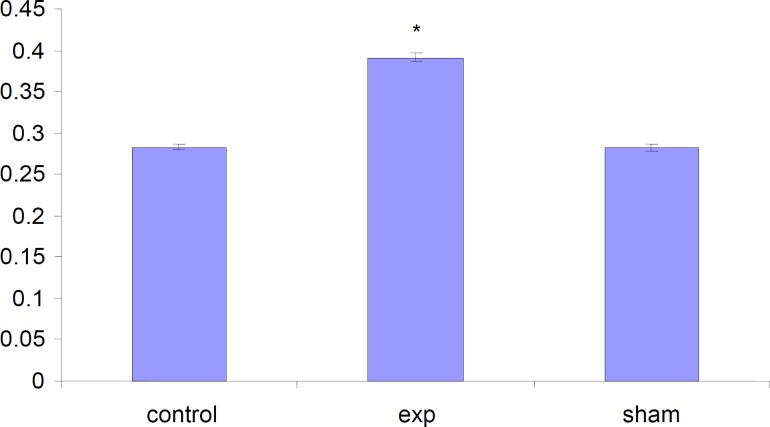
Comparison of weight of fetuses in the test, sham and the control groups. Values represented as mean±SD (n=3). * P<0.001 compared with sham and the control groups

*Alteration in the weight of placentas*

The weight of placenta was decreased in test group but this decrease was not significant (p < 0.001) compared to control and Sham groups. 

**Figure 6:**
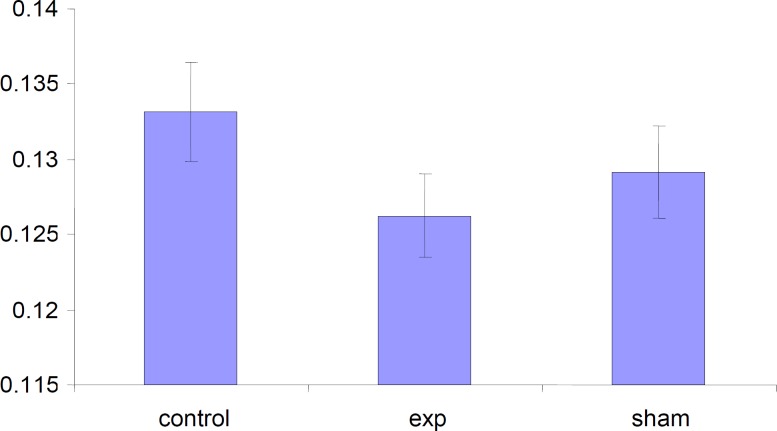
Comparison of weight of placentas in the test, sham and the control groups.Values represented as mean±SD (n=3).

*Alteration in the diameter of placentas*

The placenta diameter in test group was increased but this increase was not significant (p < 0.001) compared to control and Sham groups. 

**Figure 7:**
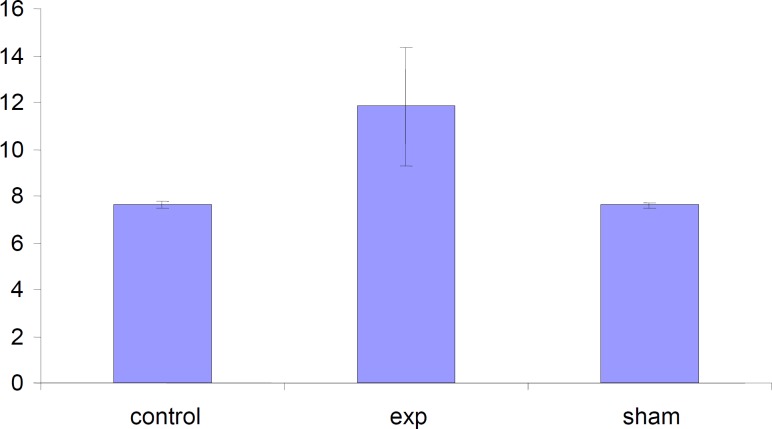
Comparison of diameter of placentas in the test , sham and the control groups. Values represented as mean±SD (n=3).

*Alteration in the length of fetuses*

The length of fetuses has significantly (P<0.001) increased in test group compared to Sham and Control groups. 

**Figure 8:**
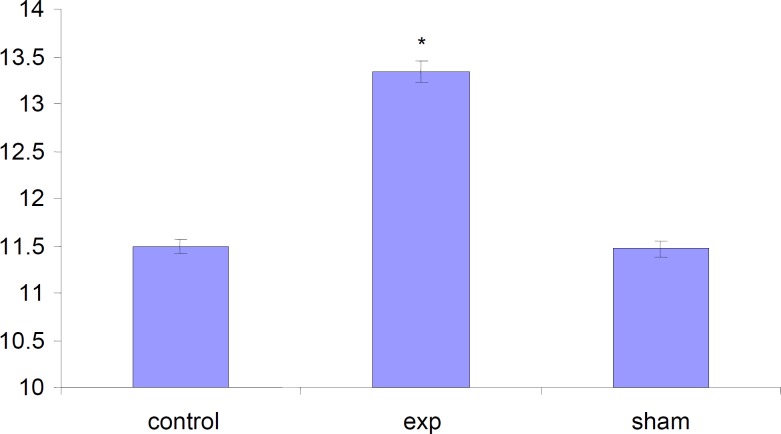
Comparison of length of Fetuses in the test, control and sham groups. Values represented as mean ± SD (n=3). * P<0.001 compared with sham and the control groups

According to Khera report in 1987 the concentrations higher than 5 mg/Kg/day of depleted uranium could cause toxicity in mouse fetus (7). One of the toxic effects of DU on mouse fetus reported in this study was decreasing in ossification of skull. In our study, the surface of cerebral cortex was straight and without curve. Domingo also reported that the Nano particles of depleted Uranium even in lower concentrations could injure the fetus brain and cause behavioral problems and mental retardation (12).

In our study we realized significant decrease in placenta weight in DU exposed test group that can be a reason for lack of sufficient blood supply to brain which leads to delay in the growth of fetus brain and probably could cause mental retardation.

In test group of our research, fetuses do not have natural C shape rotation. Domingo also mentioned this issue in his report in 1989 (8).

In this report Domingo also mentioned significant decrease in ossification of skull Demi bone, Back foot metatarsus and knuckles of front foot in the fetuses of mice already treated with uranyl acetate dihydrate (8). In our study we also found a fetus without fingers in one of its hands that is a confirmation to Domingo report (8). Domingo also reported a significant increase in liver volume and weight in fetuses of DU treated mice. 

In our study fetuses were weighted at 15 day of gestation. The weight and the length of fetuses in test group were significantly higher than those of Sham and control groups which is quite in appose to the Domingo findings in 1989 (8) , which reported significant decrease in weight of fetuses whose mothers were exposed to depleted Uranium. 

Having considered other parts of results that the liver of fetuses in test group were larger and head of them also were larger than those of control and Sham groups (Figure 4), we can conclude that our findings seem more logical than Domingo findings in 1989 (8).
